# The Week in the Courts

**Published:** 1911-01-28

**Authors:** 


### The Week in the Courts.

### UNSUCCESSFUL CLAIM AGAINST A MEDICAL PRACTITIONER.

In the Bow County Court, before Judge Smyly, a Mr. Darkins sued Mr. H. de B. Nelson, M.R.C.S., L.R.C.P., for alleged negligence in his attendance on plaintiff's wife. During the wife's confinement in March she was attended by Mr. Nelson, and complications having supervened an operation was performed by another practitioner; septic poisoning set in, and she died in May. It was alleged that defendant was negligent in his attendance, and that in consequence plaintiff was involved in extra expense. The jury returned a verdict for defendant, and judgment was entered accordingly, with costs. Mr. Matthew, on behalf of the London and Counties Medical Protection Society, Limited, appeared for the defendant.

### "THE BROWN DOG" OF BATTERSEA.

In the Chancery Division of the High Court, before Mr. Justice Neville, Miss A. L. Woodward, Hon. Secretary of the International Anti-Vivisection Council, on behalf of herself and of that Council, sought to enforce a contract made between herself and the Corporation of Battersea with respect to the erection and maintenance of "The Brown Dog Fountain" in the Battersea Recreation Ground. In accordance with an agreement the fountain was erected in 1906 at the plaintiff's expense, and certain arrangements were made to indemnify the Corporation against proceedings that might be incurred by them by reasons of the erection of the fountain. In March 1910 the defendants removed the fountain and refused to re-erect it, and the plaintiff asked the Court to order the defendants to re-erect it. His Lordship dismissed the action, holding that there was no contract by the defendants under which the plaintiff could sue. On the broader question involved: whether, if any such agreement existed, it was of a character that the Court ought to enforce by granting a mandatory injunction, his Lordship's opinion was that it was not. The inscription on the fountain was calculated to hold up University College to public execration, to inflame the public mind against it, and to lead to a breach of the peace. In dismissing the action without costs, his Lordship expressed the opinion that owing to the fluctuation of opinion of the Borough Council the plaintiff had suffered considerable hardship.

